# A Decade of mTBI Experience: What Have We Learned? A Summary of Proceedings From a NATO Lecture Series on Military mTBI

**DOI:** 10.3389/fneur.2020.00836

**Published:** 2020-08-25

**Authors:** Katherine E. Robinson-Freeman, Kassondra L. Collins, Bryan Garber, Ronel Terblanche, Marten Risling, Eric Vermetten, Markus Besemann, Alan Mistlin, Jack W. Tsao

**Affiliations:** ^1^Department of Neurology, University of Tennessee Health Science Center, Memphis, TN, United States; ^2^Department of Physical Therapy, University of Saskatchewan, Saskatoon, SK, Canada; ^3^Research and Analysis Section, Directorate of Mental Health, Canadian Forces Health Services Group, Ottawa, ON, Canada; ^4^Centre for Mental and Cognitive Health, DMRC Headey Court, Epsom, United Kingdom; ^5^Department of Neuroscience, Karolinska Institutet, Solna, Sweden; ^6^Department of Psychiatry, Brain Center Rudolf Magnus, University Medical Center Utrecht, Utrecht, Netherlands; ^7^Physical Medicine and Rehabilitation, Canadian Forces Health Services Group, Ottawa, ON, Canada; ^8^Le Bonheur Children's Hospital, Children's Foundation Research Institute, Memphis, TN, United States

**Keywords:** mTBI, concussion, military, NATO, PTSD

## Abstract

Mild traumatic brain injury (mTBI, also known as a concussion) as a consequence of battlefield blast exposure or blunt force trauma has been of increasing concern to militaries during recent conflicts. This concern is due to the frequency of exposure to improvised explosive devices for forces engaged in operations both in Iraq and Afghanistan coupled with the recognition that mTBI may go unreported or undetected. Blasts can lead to mTBI through a variety of mechanisms. Debate continues as to whether exposure to a primary blast wave alone is sufficient to create brain injury in humans, and if so, exactly how this occurs with an intact skull. Resources dedicated to research in this area have also varied substantially among contributing NATO countries. Most of the research has been conducted in the US, focused on addressing uncertainties in management practices. Development of objective diagnostic tests should be a top priority to facilitate both diagnosis and prognosis, thereby improving management. It is expected that blast exposure and blunt force trauma to the head will continue to be a potential source of injury during future conflicts. An improved understanding of the effects of blast exposure will better enable military medical providers to manage mTBI cases and develop optimal protective measures. Without the immediate pressures that come with a high operational tempo, the time is right to look back at lessons learned, make full use of available data, and modify mitigation strategies with both available evidence and new evidence as it comes to light. Toward that end, leveraging our cooperation with the civilian medical community is critical because the military experience over the past 10 years has led to a renewed interest in many similar issues pertaining to mTBI in the civilian world. Such cross-fertilization of knowledge will undoubtedly benefit all. This paper highlights similarities and differences in approach to mTBI patient care in NATO and partner countries and provides a summary of and lessons learned from a NATO lecture series on the topic of mTBI, demonstrating utility of having patients present their experiences to a medical audience, linking practical clinical care to policy approaches.

## Introduction

Over a decade ago, US medical personnel realized a high proportion of polytrauma cases evacuated from Operation Enduring Freedom/Operation Iraqi Freedom (OEF/OIF) had unrecognized TBI. These cases were primarily linked to blast-related injury due to improvised explosive devices (IEDs) being the weapon of choice in insurgency warfare (1). Many estimated that mTBI would be more pervasive because the symptoms would go unrecognized (2). Mild traumatic brain injury (mTBI) has become a growing problem in military medicine and has garnered attention recently from both the military and veteran communities. A large-scale survey by the Research and Development (RAND) corporation estimated that as many as 300,000 US military personnel who had served in OIF/OEF had sustained mTBI (3). Speculation arose that pure blast wave exposure was sufficient to create mTBI which defied conventional theories on mechanisms of head injury from blast where the intact skull would protect the brain from injury. It was also discovered that unrecognized acute mTBI, with disruption of executive functioning and reaction time, could expose the injured individual or other soldiers working with the concussed, to further injury or death in a combat setting (1, 4). Theories also arose that subsequent disability from mTBI would be long-lasting, which again defied the conventional belief that mTBI was an acute injury and that persistent symptoms (beyond 3–6 months) occurred in a very small minority of individuals (5, 6).

In 2009, the NATO Human Factors in Medicine (HFM) division gathered a working group comprised of representatives from NATO and partner nations (Canada, France, Netherlands, Sweden, United Kingdom, United States) to identify and provide recommendations for mTBI policy related to diagnosis, treatment, and rehabilitation in the military operational (deployed) setting. Efforts evolved over the years to address several issues:

To study the mechanics and pathophysiology of blast induced brain injury

To systematically study the epidemiology of blast induced mTBI

To better identify methods to screen for and diagnose mTBI

To better understand the long-term consequences of blast-related mTBI

To identify optimal strategies for the acute management of mTBI in order to determine fitness for duty in highly kinetic combat operations as well as identify optimal clinical pathways for management of persistent symptoms following mTBI.

In 2015, the group published summary recommendations. This paper describes follow-on efforts to disseminate knowledge gained through a NATO HFM-sponsored lecture series delivered in multiple countries in 2016, 2018, and 2020 (7). Each subsection below represents a specific topic discussed at the lecture events and in the summary recommendations.

## Patient Perspectives

An invaluable outcome of these conferences was the recognition of the need for involving patients and their perspectives in medical education and policy decision making. As patients shared their experiences, both researchers and clinicians were able to participate in a dialogue with patients in order to understand their perspectives on evaluation and therapies and allow their viewpoints to inform clinical practice and research. It also allowed clinicians and researchers to put their practice into a larger perspective of the patients' needs both at discreet time points in their recovery and as a part of the patients' long-term goals. An example of this is based on a patients experiences of having to recite the story of their injury multiple times for multiple providers. A joint intake session for all providers on an interdisciplinary team could allow more seamless care. Patient perspectives are essential to understanding the challenges faced by this specific population and are vital to guiding further research (8, 9).

## Operational Definition of mTBI and NATO Policies

The symptoms of mTBI typically include headaches, sleep disturbances, dizziness, slowed thinking, poor concentration, and memory problems, as well as anxiety and depression (10, 11). These symptoms may manifest in a multitude of ways, including slower reaction times, decreased energy, problems maintaining balance, difficulty multi-tasking, increased interpersonal problems, and other visibly observable personality changes. When these manifestations are seen in an operational setting, service members may experience failure to relay or recall information quickly, poor marksmanship, decreased work performance, failure to identify threats, difficulty in making rapid decisions, and deficits in other performance related duties. Such deviations from normal performance can lead to increased risk to the individual, the unit, and the entire mission at hand. The majority of people recover from a mTBI/concussion in 7–10 days, but some cases fall into the prolonged category (1–4 weeks) and into the persistent category (>3 months) with estimates of persistent post-concussive symptoms (PCS) varying between 10 and 20% of patients (12–15).

The terminology surrounding mTBI and its operational definition is up for debate. Some NATO countries prefer the term mTBI while others prefer the term concussion to be used during interactions with patients. For countries that prefer “concussion,” such as the United States, the rationale is that it emphasizes the difference between mTBI and more severe forms of TBI and it is familiar to the patient or service member from exposure to athletics and other circumstances. The use of “concussion” may make treatment and recovery feel less threatening and increase the expectation of a successful recovery. Other countries prefer mTBI because it gives a sense of validation to the pain and difficulties that service members are experiencing; that this is something beyond a normal headache.

NATO countries have variations in their specific definitions of mTBI/concussion (Table 1). Currently, 4 NATO countries have mTBI policies: USA, United Kingdom (UK), Canada, and the Netherlands, with relatively similar definitions used by each nation. The United States Department of Defense defines mTBI as a disruption of brain function from a blow or jolt to the head or penetrating head injury indicated by a new onset or worsening of at least one of the following: loss of consciousness (LOC) of 0–30 min, post-traumatic amnesia (PTA) of up to 24 h, alteration of consciousness (AOC) from a moment up to 24 h, neurological deficits that may or may not be temporary, and/or an intracranial lesion. The UK defines mTBI using the same LOC, AOC, and PTA criteria, but has added the possibility of transient neurological abnormality and a Glasgow Coma Scale (GCS) score of no >13, 30 min post-injury. Canada Forces Health Services defines mTBI as a head injury event and alteration of consciousness, which includes the possibility of PTA and/or LOC (16). The Netherlands defines mTBI as a closed head injury followed by a post-traumatic amnesia lasting <1 h and/or loss of consciousness lasting <15 min (17). A consensus definition between NATO countries would facilitate consistent universally accepted care, availability of shared diagnostic tools and clinical management strategies, as well as allow for easier comparison of research findings (7). However, it is important to note that certain characteristics of mTBI stated in the definitions above can also occur with certain anxiety-related psychological conditions, which are often comorbid with mTBI, thus making the application of a common definition more difficult (18).

**Table 1:** Definition on mTBI/concussion from various NATO countries.

**Country**	**LOC**	**PTA**	**AOC**	**GCS**	**Additional**
USA	0–30 min	Up to 24 h	Up to 24 h	NA	Permanent/transient neurological defects possible intracranial lesion
UK	0–30 min	Up to 24 h	Up to 24 h	No <13 at 30 min post injury	Possible transient neurological abnormality
Canada	Possibility	Possibility	Yes	NA	Must include head injury event
Netherlands	<15 min	<1 h	NA	NA	Closed head injury

## Blast-Related mTBI

The concept that pure blast wave alone could injure the brain enclosed in the intact skull first arose in the First World War and was termed “Shell Shock” (19). The term “Shell Shock” was initially used by the soldiers directly affected by such blast exposure to describe symptoms such as fatigue, tremor, confusion, nightmares, impaired sight and hearing, loss of balance, and impaired sensation, in which there was no visual explanation for the symptoms (20). “Shell Shock” was distinguished from PTSD due to the physical symptoms that prevailed. However, the first few cases described were deemed to be psychological issues that stemmed from repressing the memories of the blast trauma (20). The initial treatment routine involved, quick treatment, being treated away from the combat field, and psychotherapeutic measures (20). Although we have come a long way in understanding these symptoms that arise from blast exposure, the prevailing scientific belief since then has been that there is no plausible mechanism by which brain injury can occur. However, we have seen a resurgence of this idea that has sparked much controversy amongst healthcare professionals and researchers: can pure blast alone cause mTBI directly? One case study, conducted in 2009 described a service member with a mTBI that resulted from primary blast wave alone, possibly supporting the theory that blasts alone can cause mTBI (21).

Blast exposure can cause injury through several different mechanisms. Primary effects include injuries related to the supersonic pressure changes in a very short amount of time. Secondary effects tend to be caused by shrapnel and other flying objects moved by the blast, which can cause penetrating or blunt force impact injuries. Tertiary effects involve acceleration of the body caused by the physical blast itself, which can result in tissue shearing and diffuse injuries within the brain. Quaternary effects are caused by heat, smoke, or emission of electromagnetic pulses (22). Often mTBI can be due to one or more of these effects (23). A number of experimental models have been developed in order to evaluate the relative importance of the primary, secondary, tertiary and quaternary blast injury mechanisms (24, 25). Classical mTBI in sports medicine is often mediated by an acceleration/rotational injury mechanism, which could correspond to tertiary blast injury. Experimental rotational TBI, at acceleration levels that do not cause neuronal cell death or focal injury, results in limited white matter injuries (26), increases in injury serum biomarkers such as S100B and a transient memory impairment (25) which is a picture that could be argued to resemble a clinical mTBI. An experimental mild exposure to primary blast is unlikely to induce neuronal cell death or white matter injuries, but has been observed to induce changes in certain transmitter systems including the projections from locus coeruleus and the dorsal raphe nucleus to brain regions such as prefrontal cortex, hippocampal formation and the hypothalamus (27). Changes in monoamine neurotransmitters such as noradrenaline and serotonin could have implications for mood changes and other persistent symptoms following mTBI. Thus, experimental studies implicate that both primary and tertiary blast could induce functional changes in the brain that may be assumed to be relevant for the changes that are observed in real life mTBI.

An interesting area of research concerns breachers, those tasked with entering buildings or rooms via explosive chargers resulting in repeated blast exposure. Carr et al. reported that there were differences in neurocognitive testing in the instructors who teach breaching compared to the students, suggesting that there may be negative effects of repeated blast exposure (28). Studies have found that trainers rather than trainees show evidence of more neurological impairment than expected in unexposed populations (29). Breachers represent a population exposed to repeated low-level blast that could be important in further understanding the controversy surrounding pure-blast mTBI (18, 28, 29).

Blast-related mTBIs were prevalent in theater, accounting for 68% of 433 United States casualties from OIF/OEF treated at the former Walter Reed Army Medical Center, Washington, DC from 2003 to 2005 (30). Diffusion tensor imaging (DTI), a form of magnetic resonance imaging, is sensitive to diffuse axonal injury and small hemorrhages that occur with blast related mTBI. However, studies have not consistently shown DTI abnormalities in patients with chronic symptoms. Studies have shown abnormalities at 6–12 months post injury, but other studies have found no abnormalities at 2 or more years post injury with DTI, although patients were still experiencing post concussive symptoms, cognitive defects, and PTSD (31–36). Schneiderman et al. reported that 5 months post deployment blast-related and non-blast-related mTBI patients did not differ in experiencing persistent symptoms (37). However, another study reported better performance in participants with mild blast-related mTBI, but poor performance for participants who experienced moderate-to-severe blast-related mTBI on a visual memory test when compared to non-blast related mTBI participants (38). Schneiderman also reported a marginal increase in PTSD symptoms for blast-related participants (37). A similar study examined blast and blunt force concussions using the Automated Neuropsychological Assessment Metrics (ANAM) test (39). There were no significant differences in scores at the initial assessment, 10 days after injury, or at 15 days after injury. MacDonald et al. found similar results when comparing blast-plus-impact mTBI to non-blast-related mTBI (Macdonald JAMA neurology 2014). Overall, there seem to be few quantifiable differences between blasted-related mTBI and non-blast-related mTBI.

## Epidemiology of mTBI

In the Iraq and Afghanistan wars, injuries to the head and neck were identified in 22% of wounded Service members evacuated from the combat theater, reportedly more compared to the 12–14% of combat casualties in the Vietnam War designated as brain injuries (40, 41). The increase in diagnosed brain injuries can be attributed to several factors, including: better medical care allowing Service members with polytrauma to survive, improvements in personal protective equipment, and the rise of IEDs as a preferred weapon (40, 41). The prevalence of mTBI can be difficult to estimate in both civilian and military populations due to the complexity of definitions and standards used to identify traumatic brain injuries (42–44). The incidences may be captured by looking at medical records or by self-reports, both of which have their biases. Medical records may not capture people who did not or could not seek treatment; additionally their results may have been skewed by billing code errors (44, 45). Self-reports are reliant on the memory and cooperation of participants' ability to report the incident accurately (46–48). One group who reviewed 121 different studies of mTBI suggested the incidence to be >600 per 100,000 persons (49). It is also important to note that the US has a higher rate of mTBI than the world averages, while the UK and Canada have decidedly lower rates (50, 51). The length of deployment implemented by each of these countries drastically differs and may affect the variation in the incidence rates of mTBI (50, 51). In combat situations, Service members often do not seek medical treatment after an injury in order to continue the mission and may even be encouraged to dismiss symptoms (18). Some NATO nations (USA and Netherlands) have begun to employ event-based screening. Event based screening requires all personnel affected by an event to report for immediate testing. Such practices may become the gold standard for military identification and diagnosis of mTBI (18).

## Acute mTBI Management

Acute phase of a mTBI is characterized by a transient disruption in consciousness associated with an exposure to an injury mechanism. Physical symptoms such as headache, dizziness, and cognitive impairments may follow (11). In military settings, individuals are often exposed to blast events that cause not only mTBI but also physical injuries and psychological trauma that may mimic or coincide with mTBI. Often soldiers are reluctant to seek care and the high operation tempo of combat zones make return to duty decisions challenging (52). Primary goals of acute care management are to: identify more serious cases of intracranial injury that may require urgent neurosurgical evaluation; facilitate early care seeking for all possible cases of mTBI; expectant management of symptoms and return to duty determinations (47, 48, 53, 54).

### Acute Evaluation

Debate continues about the optimal method of promoting care seeking for suspected cases of mTBI. Both the UK and Canada employ a symptom-based approach for identifying Service members who have been concussed or sustained a mTBI, while the United States and the Netherlands have an event-based approach. The United States requires all military personnel within 50 m of a blast or potentially concussive event to undergo medical evaluation, while the Netherlands uses a 25-m distance (18). Both distances were set fairly arbitrarily with the intention to evaluate all involved and be able to better diagnose injury following the results of the initial medical evaluations. Active case finding could be advantageous because the published sports literature has suggested that competitive athletes often avoid seeking care, which may be extrapolated to the military population and a sense of not wanting to let the team/unit down (55). The weakness of the current active case finding procedure is the need for identification of the true risk of injury relative to the proximity of an IED blast and a cost/benefit analysis (18). The difficulty in testing procedures and active vs. passive case finding highlights the need for an objective, sensitive, reliable and field-worthy bedside test (16).

Acute evaluations of uncomplicated mTBI cases typically involve assessment of symptoms and signs. Several NATO countries have implemented clinical algorithms and clinical practice guidelines for in-theater management of mTBI. Almost all of these have been adapted from existing guidelines in the sports concussion literature and have never been rigorously evaluated in the acute combat or operational setting (16). Initial evaluation of a head injury must assess for a possible more serious intracranial injury (16). Many clinical practice guidelines include “red flags” that may suggest something more serious than a concussion has occurred (16). The Ottawa Rules are a set of guidelines for concussion diagnosis that includes high risk patients that have a Glasgow Coma Scale (GCS) score of <15 at 2 h post injury, a suspected open or depressed skull fracture, a basal skull fracture, more than 2 episodes of vomiting, or patients over 65 years old (56, 57). The New Orleans Rule includes much of the same: headache, vomiting, older than 60, drug or alcohol intoxication, persistent anterograde amnesia, visible trauma above the clavicle, and seizure (56, 57). Again, it is important to note that both were developed for civilian use and may not extrapolate well to operational settings (58).

The presence of neurocognitive deficits, such as trouble concentrating and multi-tasking as well as memory problems, can indicate the severity of an injury and allow healthcare professionals to track recovery. Traditional testing included written tests, but in the past two decades there has been a shift to automated, computerized testing such as the ANAM, Immediate Post-Concussion Assessment and Cognitive Testing (ImPACT), and Defense Automated Neurobehavioral Assessment (DANA) in use by the US military (59, 60). However, these computerized tests must complement, not substitute, a clinical examination (61). NATO nations differ in the types and timing of the neurocognitive testing used. The US and Canada require using the Military Acute Concussion Evaluation (MACE), while the UK uses a self-report symptom measure to monitor symptom resolution called the Rivermead post-concussion questionnaire (RPQ) (16). The US and Canada are the only nations that use exertion based testing to make return-to-duty determinations (16). The US performs post-injury neurocognitive testing routinely, while other nations perform it on a discretionary basis (16). The US also requires pre-deployment baseline neurocognitive testing with the intent of having data which can be used for comparison purposes after an injury (16). There are several concerns with this policy such as the reliability of this baseline, which depends on the context of testing, practice effects, personal effort, and cost/benefit analysis. Practice effects occur when repeat testing familiarizes patients with the content of the test, such as words used in memory components. Thus, a Service member performs better in subsequent testing due to a learning effect (62, 63). However, multiple versions of a test may counteract this effect (62, 63). Practitioners have also expressed concerns that Servicemen may intentionally perform poorly on the baseline assessments in order to allow them to pass with ease in the future. Cost/benefit analysis would review how often the post-deployment screening revealed a TBI when compared to baseline while accounting for the cost of the policy; early reports have noted that the number of pre-deployment screenings used for comparison after a post-injury event has been extremely low. Overall, computerized tests have only a moderate reliability and validity and the policy of pre- and post-deployment screening has yet to be scientifically proven.

It should be re-emphasized that early detection of mTBI/concussion is critical to achieve symptom resolution as rest (physical and cognitive) and education (managing expectations) are the only Level A evidence for concussion treatment (47, 48, 53, 54). There are certain activities within the acute phase that help brain recovery such as maximizing rest, adequate sleep, keeping a low heart rate, avoidance of heat, and limiting physical activity. Early education is also noted to assist in progressive return to activity following mTBI. In addition, there are certain activities than can hinder brain recovery such as mental exertion, inadequate sleep, caffeine use, physical exertion, and a second concussion. Avoidance of aspirin and non-steroidal pain medications in the first 48 h is recommended due to an increased risk for bleeding.

### Progressive Return to Duty

A progressive return-to-duty is recommended by all NATO nations, beginning with a 24-h rest period following the time of injury. From this basic policy, individual counties vary on what defines a progressive return to duty (Table 2) (41). Before returning to duty, the US and Canada require patients to be asymptomatic with a MACE score >25/30 after exertion based testing, while the UK bases decisions on RPQ scores and may or may not include exertion based testing. Elements to consider in making return-to-duty decisions are severity and number of clinical symptoms, physical examination, concussion history, and whether symptoms can be provoked by exertion-based testing in an asymptomatic Service member. Exertion based testing allows healthcare providers to assess how the patient may function in an operational setting where physical exertion is often a part of daily duties. For exertion based testing, patients should exert to 65–85% of target heart rate and maintain this for 2 min (64). Healthcare providers may also recommend pacing principles, lifestyle changes such as caffeine consumption and diet modifications, cognitive strategies for energy conservation, and activity scheduling to help with progressive return-to-duty. However, a recent study suggests that exercise and activity within the first 7 days post-injury is associated with lower risk of PCS at up to 28 days post-injury in children and adolscents (65). It is important to note that this study would need to be replicated in an adult military population and include a longer follow-up period if it were to impact return-to-duty guidelines.

**Table 2:** Progressive return to duty guidelines for various NATO countries.

**24 h rest period for all countries**		
**UK** • RPQ scores • Possible exertional testing	**USA/Canada** • Asymptomatic • MAE >25/30 after exertional testing	**Additional considerations** • Severity & number of clinical symptoms • Physical exam • Concussion history • Exertional testing

*RPQ, Rivermead Post Concussion Symptoms Questionnaire*.

## Management of Symptoms

It should be noted that other than rest and education, there are no “concussion-specific” treatment modalities and that all treatment recommendations stem from the appropriate algorithm for specific conditions found in various guidelines and care maps. Clinicians are encouraged to “categorize” symptoms and symptom clusters into existing treatment guidelines for those conditions and treat appropriately (66). Regrettably this is often a trial and error approach as every concussion patient will respond somewhat differently.

### Headache

Acute headache management can consist of acetaminophen, if headaches persist and evolve into a migraine form (52), Triptan agents can also be used (67). For persistent headaches, low doses of tricyclic antidepressants, such as amitriptyline, can be used nightly (68, 69). Sleep problems can be managed using medications such as zolpidem, with emphasis to be placed on getting sufficient nightly sleep (52). Treatment of vertigo consists of determining the source of the symptoms and may require canal repositioning maneuvers (52).

### Vestibular Symptoms

Vestibular disturbances are sequelae of mTBI/concussion in ~30% of patients. These disturbances include benign paroxysmal positional vertigo, exercise-induced dizziness, migraine-associated dizziness, and spatial disorientation. Neurological exams (Dix Hallpike test and others) can help to elucidate what symptoms the patient is experiencing and their origin. Treatment can include vestibular physical therapy, Cawthorne-Cooksey exercises, which are progressive exercises to combat vertigo, and gaze stabilization exercises with improvement expected after 72 h post-injury (52).

### Endocrine Dysfunction

Endocrine dysfunction is also present in ~40% of all mTBI patients, likely due to damage to the hypothalamic-pituitary brain structures. Symptoms can include depression, anxiety, fatigue, poor memory, decreased concentration or libido, infertility, weight loss/gain, reduced heart rate or blood pressure, and hair loss. These symptoms often are mistaken for other TBI-related sequelae. If a patient's symptoms do not resolve within 3 months and are suggestive of an endocrine dysfunction, laboratory tests should be performed to examine various hormone levels that may be affected (TSH, cortisol, LH, FSH, Prolactin, IGF). If hormone levels are abnormal, endocrinology referral should be considered. Endocrine dysfunction after mTBI is often unrecognized, thus more routine hormonal screening of mTBI patients is necessary. However, additional studies are needed to determine the true incidence of such abnormalities (52).

### Sleep Disruption

Sleep disruption is another problem encountered by mTBI patients and can take the form of either insomnia or hypersomnolence. The problem of sleep disruption is also complicated by the presence of comorbidities such as PTSD and pain. Again, obtaining a thorough history is important to understanding the patient's sleep quality before and after the injury and to discuss their current sleep hygiene and routine. Treatment may include education about sleep hygiene, relaxation techniques, and non-benzodiazepine hypnotic medications. Sleep hygiene is most important to reinforce with patients suffering mTBI as rest is the most important part of clinical recovery within the acute phase of recovery. Patients should be encouraged to reduce caffeine and alcohol intake, not to clock-watch, to turn off all electronic devices and to establish a good “wind-down” routine) prior to bedtime (52).

### Visual Complaints

Ninety percentage of patients with a concussion suffer an issue with eye function (70). As vision is a function of the brain and over half the brain's circuit are dedicated to vision and eye movements, visual disturbances and changes in function may be useful in diagnosis of mTBI. Defects in the visual system can occur in both the efferent and afferent pathways of the brain. Defects in the afferent system can include decreased acuity, color differentiation, contrast sensitivity and decrease pupillary light response if there is direct trauma to the optic nerve (71–73). Midline shift and visual attention deficits may also occur (71, 72). Hyperactivity in the orbitofrontal region and right hippocampus may cause abnormalities with visuoperceptual scene processing, visual working memory, and visual attention efficiency (74). Efferent visual dysfunction often includes decreased accommodation and convergence amplitudes, diplopia, slowed pupillary reactions, and a reduction in pursuit ability (72). Reading impairment is also common and can last 6 months post-injury and even longer in older patients (70). Visual changes may be able to serve as a biomarker for concussion with several testing procedures available, such as rapid sideline detection and the King-Devick Test (75, 76).

## The Impact of mTBI on Families

Another aspect of care for patients with mTBI is the role of their family, which is often viewed as a secondary concern, but which should be focused upon as an extension of rehabilitative care. Service members coming home with an injury may have difficulty reintegrating into civilian life and the family may have trouble adjusting to a “new normal.” It is important that the patient get expert and early follow up care (47, 48, 53, 54). The family needs clear and accurate information, beyond just a leaflet, to facilitate a better understanding of mTBI and how they can assist their family member. Patients need collaboration and integrated care that includes both health care professionals and a family dimension. The education should focus on cognitive symptoms, behavioral symptoms, physical rehabilitation as well as comorbid disorders e.g., PTSD. It is also important for patients and family members to acknowledge this reconstruction of the family in order to recognize positive changes such as “growing together” and manage expectations of both the patient and family members.

## Comorbid Disorders

The issue of co-morbidities appears more pronounced in populations with an existing diagnosis of a traumatic brain injury. For example, among service members with a history of mTBI, two large studies found PTSD prevalence is at 33–39% of service members. Lew found that in a treatment-seeking sample of 340 VA eligible service members, 81.5% reported chronic pain symptoms, 68.2% reported PTSD symptoms, 66.8% reported TBI symptoms and 42.1% reported symptoms of all three (67). These are now known as the triad of co-occurring conditions with mTBI. Additional symptoms included sleep disorders, substance abuse, psychiatric illness, vestibular disorders, visual disorders, and cognitive disorders. The co-morbidity of PTSD with a history of mTBI, chronic pain and substance abuse is common in the military and complicates recovery from any single condition.

Several studies from various NATO nations have indicated that there is a strong association between PCS and psychiatric illnesses, such as PTSD and depression (77). Additional studies have noted that many, if not most, PCS appeared to be strongly related to or influenced by comorbid PTSD (37, 50, 51, 78). The most common overlapping symptoms are sleep disturbances and irritability. Sleep disturbances may drive the presence of other complicating symptoms, which may provide grounds for treating it as the primary focus of interventions. The overlap between the two diagnoses strengthens the argument for using a multi-disciplinary approach for treating mTBI, especially for those with persistent symptoms. However, flashbacks are not a symptom of mTBI and neurocognitive problems, aside from memory deficits, are not often a symptom of PTSD. When controlling for PTSD and depression, the only symptom significantly correlated with mTBI is headache (78). Other studies have shown that after controlling for shared symptoms by removing them from the PTSD score, the strongest factor associated with PCS was PTSD (37). Also, studies have shown that the prevalence of PTSD is higher in patients who have sustained a mTBI than those who did not (79). Additionally, Cooper et al. reported that Service members with high levels of combat-related stress had a 3–8-fold increase in PCS (53). It should also be noted that some studies have found that blast-related mTBI has been associated more with re-experienced symptoms than non-blast-related symptoms. This study also found that the two groups of patients with mTBI did not differ in regards to other PTSD symptoms (80). The question remains as to whether the mTBI causes the psychiatric illness or the psychiatric illness just complicates the diagnosis and recovery of mTBI. These two studies further established a need for a gold standard diagnostic test for both mTBI/concussion and PTSD as well as longitudinal studies with control groups. Early detection and evidence-based treatment for both conditions may lead to better outcomes for patients. However, the complications of treating mTBI along with PTSD, multiple injuries, etc., further reinforce the need for a multidisciplinary approach to rehabilitation (15).

## Long-Term Consequences and PCS

PCS refers to symptoms of a mTBI lasting longer than 3 months after the initial injury (47, 48). In military settings, the estimates of patients with PCS vary based on country with 33% in the US and 25% in Canada (15). If a patient is not recovering after standard care, it is important to ensure all aspects of concussion care are being addressed, with the possibility of pursuing a multi-disciplinary approach to further evaluation and treatment. There are few predictors of PCS and the diagnosis is often further complicated by unrecognized and recognized psychiatric diagnoses and medically unexplained physical symptoms.

Risk factors for developing PCS headaches include military combatants, gender, and prior headache history (81–83). However, it should be noted that there is a significant disparity in prevalence in different countries because of social, ethnic, and cultural factors (84, 85). Risk factors for developing long term sequelae include: multiple mTBIs, mTBIs received before initial recovery is complete, mTBI overlapping with PTSD or anxiety, pain/fatigue, incentives for exaggerating symptom reporting, sleep issues, depression, and mTBIs resulting from close blast exposure (18).

Symptom expression and management is unique to every individual diagnosed with a concussion/mTBI. However, there are best practice guidelines available that attempt to provide the most adequate holistic care to each patient and help prevent PCS. Lasting headaches are among the most common PCS and often resemble migraines, presenting with head pain, visual changes, nausea, irritability, and decreased ability to concentrate. Referral to a specialist becomes necessary with the sudden onset of the patient's worst headache of their life, severe eye pain, vomiting, double vision, or a change in mental status. It is important that healthcare professionals obtain a thorough medical history to ascertain the headache features, including duration, location, and frequency, which will allow more individualized treatment. Several options exist for treatment including NSAIDs, migraine medicines, tricyclic antidepressants, Botulinum toxin chemodenervation, and combination therapy. In addition to treating the patient's symptoms, it is necessary to educate the patient as to the impact of excess caffeine use, analgesic overuse, relaxation techniques, and what to do if symptoms continue to worsen (52). It is also important to note that persistent symptoms are often intertwined with comorbid mental health diagnoses such as PTSD. It can be difficult to determine the root cause of the symptoms, and thus more difficult to treat. These comorbidities also further emphasize the need for holistic treatment of the patient.

One of the most severe long-term consequences of TBI is Chronic Traumatic Encephalopathy (CTE), which has been observed in American football and ice hockey players. CTE refers to a specific degenerative brain disorder that is characterized by deposits of tau protein. It has been associated with various types of repetitive head trauma, most frequently with contact sports. The mean length of exposure to repetitive head trauma in CTE cases is around 15 years (86). For research purposes, the latency between injury and onset of clinical symptoms makes it difficult to model in rodents (24). Physical impacts to the head are probably necessary to induce injury that might lead to CTE, while side pressure from weapons or repeated blast exposure seem less likely to induce CTE. Also, studies involving breachers do not seem to suggest a relationship between repeated blast exposure and CTE (28, 87). Multiple mTBI may be a risk factor for CTE but the fact that many of those with repetitive head injuries do not develop this outcome highlights our lack of understanding about any given individuals ability to undergo brain healing after injury. The 2012 National Consensus Statement agreed that there is a possibility of long term effects due to repeat concussive events, however, CTE specifically is not well-understood and the incidence within athletes is still unknown (88). Importantly is has also been established that there is no clear connection between the cause and effect of CTE and repeat concussive events to date (88).

Currently the only factors known to contribute to PCS in civilian populations are PTSD and ongoing litigation (89). Following deployment to Iraq or Afghanistan, PCS estimates ranged from 25 to 33%, which is higher than the civilian estimates (15). Veterans of combat in OIF/OEF who screen positive for TBI at VA medical centers, after returning from deployment, had higher rates of neurological deficits and PTSD with increasing numbers of LOC events (90). However, most of the studies on PCS are retrospective and based on self-reports; well-conducted prospective studies with an objectively identified injury event are needed. Further research is also needed to determine if there is increasing risk of PCS and chronic symptoms after multiple mTBI events (15). It is important to note that the majority of military mTBI cases recover with no long term sequelae.

## The Next Decade of mTBI Research

There are several challenges for the research domain of mTBI. One of the biggest challenges facing mTBI research and clinical practice is the need for a gold standard diagnostic test. This test would need to be valid, objective, and reliable. However, the uniqueness of military operational settings also requires that the test not only be easily administered and interpreted, but also useable in diverse and extreme environments. Additionally, this test would need to be specific to situations where polytrauma or acute stress may result from a life threatening combat event (7). There are several candidates that could fulfill these requirements worthy of investigation: blood biomarkers, electrophysiological tests, measurements of cerebral blood flow and intracranial pressure, neurocognitive assessment, and sensory assessment. In particular, there has been a recent focus on the search for blood biomarkers (91, 92). The development of this diagnostic test would also allow for earlier detection, which could lead to improvement of treatment outcomes.

In addition to the need for a gold standard diagnostic test, each one of the controversies discussed above requires further research to definitively state the best policy for military operations. A universal definition would allow nations to work more collaboratively on teasing out the mechanisms and best treatments for mTBI. Increased understanding of the value of pre- and post-deployment screening and active case finding would allow militaries to better identify personnel who may be at risk for mTBI. A clear understanding of the interplay between PTSD and mTBI would allow clinicians to improve patient therapies. Overall, there is a general need for more research into mTBI/concussion especially considering how incidence has risen in recent years and more Service members are being affected.

Research using animal models for mTBI is also needed in order to gain a more thorough understanding of the etiology of the condition. The model could be selective to examine different contributing factors to mTBI such as primary blast, penetrating fragments, acceleration movements and a combination of these. Animal models that are well-documented could also provide an avenue for comparison between research done in different laboratories and lead to an increase in knowledge about mTBI. Several procedures could be used to study mTBI in animals, such as open field exposure, blast tubes for explosives, shock tubes with compressed air or gas, and models for penetrating injuries as well as acceleration and decelerations. However, translation between real life clinical situations and animal models present several problems. The timetable for injuries and symptoms can be assumed to be different for humans compared to rodents. It is also difficult to assess post-concussive symptoms like headache or mood changes in rodents. LOC is difficult to evaluate in anesthetized animals and rodents do not have the capability to vomit in the same way as humans. However, there are methods to evaluate anxiety and sleep pattern changes, which should be of relevance. Specific studies on vestibular functions or transmission in pain related pathways could also appear as relevant areas of research for mTBI in rodents. Methods such as imaging, including functional MRI, or serological biomarkers should also be of clear value for the translation between clinical and experimental mTBI. Experiments including repeated blast exposures or other types of mTBI could also create a relevant correlate to studies on breaching.

It is important for those working with military populations to use the evolving focus on scientific research on sports concussion and pediatric populations to inform their practice insofar as this is possible. Researchers, especially those working with military populations, should also look to determine tools for early identification of those at risk of poor long-term outcomes. A better understanding of the physiology of brain healing is also necessary, which may be gained in animal research, and will help inform studies looking for better treatments. Several other gaps in research knowledge that affect military populations include: gender differences, the effect of age, repeat injuries, and long term consequences, as well as the influence of diet on brain inflammation and healing (93).

As the nature of military operations change, exposure to blast and risk of mTBI will fluctuate. However, there has been a resurgence of interest amongst medical experts to further research these questions in the field of sports medicine and pediatrics. The opportunity to share knowledge and advance critical areas of research will likely continue between military and civilian scientists and medical experts to hopefully resolve many of these outstanding knowledge gaps.

Identifying a biomarker that is easily obtained and determines the presence and severity of a head injury would the next ideal step to managing traumatic brain injuries. Research has been conducted on proteins, microRNA's, and metabolites as potential biomarkers; however, none seem to be living up to the gold standard expectation (94). S100B has been the most research-focused protein to be used as a biomarker for mTBI (95). Naturally this protein is found in the cerebral spinal fluid, and is expressed within astrocytes (94). Levels of S100B have been shown to become elevated within the cerebral spinal fluid after the injury of these astrocytes, due to moderate to severe brain injury events (95). Unfortunately S100B levels are difficult to accurately determine due to the ineffectiveness of the protein to cross the blood brain barrier, and are also elevated with other orthopedic injuries (96). Using microRNAs as biomarkers has also been difficult due the differences in expression based on the severity of the trauma (97). For instance, miR-765 levels did not vary in those with a mTBI, while levels of miR92a and miR-16 were seen to increase after injury (97). However, after a severe TBI levels of miR-16 and miR-92a were decreased, and levels of miR-765 were increased (98). Using multiple microRNA expression level testing may help to combat this issue and allow for determination of the severity of the injury (94). Using changes in metabolism may also be a promising way to detect the severity or presence of an injury, currently N-acetylaspartic acid (NAA) is the most promising option. A study found that levels of NAA decreased throughout the whole brain after a mTBI, even if there were no signs of the injury in an MRI (99). Additionally another study found that NAA levels were decreased in athletes who had been diagnosed with a concussion 3 days prior to testing. The NAA levels returned to those of controls by 30 days post injury (100). Other biomarkers under investigation include NF-L, NF-H, GFAP, UCHL1, NSE, and MBP. NF-H levels have been increased in children with acute TBI who had worse GSC and some studies have found similar results in adults. GFAP levels have been correlated with severity across levels of TBI. The predictive value of GFAP is increased when combined with UCHL1 levels, as UCHL1 is not CNS specific and therefore is limited in its use individually. Tau proteins were also found to be correlated with severity of mTBI self-reported symptoms, but these difference were small, sometimes even below levels of detection of certain devices (101). Increased levels of GFAP and UCHL1 have also been noted post trauma in patients enrolled in the CARE cortsium, a partnership between the NCAA and the Department of Defense in the United States (102). The FDA has approved a blood test to predict the presence of intracranial lesions indentifiable on CT scan using UCHL1 and GFAP levels, The Brain Trauma Indicator, after reviewing a clinical trail showing the sensitivity to be 97.6% and the negative predictive value to be 99.6%. However, the positive predictive value in those with GCS of 14–15 was only 8.8% (103). An additional study used breakdown products of GFAP, GFAP-BDP, in its assay, and found that with a cut off value of 0.68 ng/mL the test has a positive predictive value of 87% with a sensitivity of 73% and a specificity of 89% (104). The use of biomarkers seems promising, however much more research is needed on the ability to determine presence of an injury and overall severity with significant accuracy. Biomarkers may also one day be able to assist in return to play standards as well as diagnoses (94).

Where possible, clinical diagnosis and management strategies should be evidence-based, and where lacking, guided by a judicious approach commensurate with the level of risk. Each NATO member country needs to explore the magnitude of this issue within the context of the scope of their own military operations before deciding on an approach that best suits their circumstances. Whatever approach is ultimately adopted needs to be balanced, logical, feasible and based on the best available scientific evidence. Continued vigilance is required to identify compelling new evidence that would warrant changes in practice.

## Author Contributions

KC and KR-F wrote the manuscript. All other authors edited the manuscript.

## Conflict of Interest

The authors declare that the research was conducted in the absence of any commercial or financial relationships that could be construed as a potential conflict of interest.
